# Determining the Agreement Between an Automated Respiratory Rate Counter and a Reference Standard for Detecting Symptoms of Pneumonia in Children: Protocol for a Cross-Sectional Study in Ethiopia

**DOI:** 10.2196/16531

**Published:** 2020-04-02

**Authors:** Charlotte Ward, Kevin Baker, Sarah Marks, Dawit Getachew, Tedila Habte, Cindy McWhorter, Paul Labarre, Jonathan Howard-Brand, Nathan P Miller, Hayalnesh Tarekegn, Solomie Jebessa Deribessa, Max Petzold, Karin Kallander

**Affiliations:** 1 Malaria Consortium London United Kingdom; 2 Department of Public Health Sciences Karolinska Institutet Solnavägen Sweden; 3 Malaria Consortium Addis Ababa Ethiopia; 4 United Nations Children’s Fund Supply Division Copenhagen Denmark; 5 United Nations Children’s Fund Programme Division New York, NY United States; 6 St Paul’s Hospital Millennium Medical College Addis Ababa Ethiopia; 7 School of Public Health and Community Medicine Institute of Medicine University of Gothenburg Gothenburg Sweden

**Keywords:** pneumonia, diagnostics, child, respiratory rate, Ethiopia

## Abstract

**Background:**

Acute respiratory infections (ARIs), primarily pneumonia, are the leading infectious cause of under-5 mortality worldwide. Manually counting respiratory rate (RR) for 60 seconds using an ARI timer is commonly practiced by community health workers to detect fast breathing, an important sign of pneumonia. However, correctly counting breaths manually and classifying the RR is challenging, often leading to inappropriate treatment. A potential solution is to introduce RR counters, which count and classify RR automatically.

**Objective:**

This study aims to determine how the RR count of an Automated Respiratory Infection Diagnostic Aid (ARIDA) agrees with the count of an expert panel of pediatricians counting RR by reviewing a video of the child’s chest for 60 seconds (reference standard), for children aged younger than 5 years with cough and/or difficult breathing.

**Methods:**

A cross-sectional study aiming to enroll 290 children aged 0 to 59 months presenting to pediatric in- and outpatient departments at a teaching hospital in Addis Ababa, Ethiopia, was conducted. Enrollment occurred between April and May 2017. Once enrolled, children participated in at least one of three types of RR evaluations: (1) agreement—measure the RR count of an ARIDA in comparison with the reference standard, (2) consistency—measure the agreement between two ARIDA devices strapped to one child, and (3) RR fluctuation—measure RR count variability over time after ARIDA attachment as measured by a manual count. The agreement and consistency of expert clinicians (ECs) counting RR for the same child with the Mark 2 ARI timer for 60 seconds was also measured in comparison with the reference standard.

**Results:**

Primary outcomes were (1) mean difference between the ARIDA and reference standard RR count (agreement) and (2) mean difference between RR counts obtained by two ARIDA devices started simultaneously (consistency).

**Conclusions:**

Study strengths included the design allowing for comparison between both ARIDA and the EC with the reference standard RR count. A limitation is that exactly the same set of breaths were not compared between ARIDA and the reference standard since ARIDA can take longer than 60 seconds to count RR. Also, manual RR counting, even when aided by a video of the child’s chest movements, is subject to human error and can result in low interrater reliability. Further work is needed to reach global consensus on the most appropriate reference standard and an acceptable level of agreement to provide ministries of health with evidence to make an informed decision on whether to scale up new automated RR counters.

**Trial Registration:**

ClinicalTrials.gov NCT03067558; https://clinicaltrials.gov/ct2/show/NCT03067558

**International Registered Report Identifier (IRRID):**

RR1-10.2196/16531

## Introduction

Acute respiratory infections (ARIs), primarily pneumonia, are the leading infectious causes of death among children aged younger than 5 years globally, accounting for an estimated 0.9 million deaths in 2015 [1], with over 75% of these deaths clustering in sub-Saharan Africa and Southeast Asia. Deaths from pneumonia in children result mostly from delayed presentation to appropriate health care providers and inappropriate treatment [2].

Diagnosis of pneumonia by community health workers (CHWs) and first-level health facility workers (FLHFWs), collectively known as frontline health workers, is based on counting the number of breaths in 60 seconds in children aged younger than 5 years with cough and/or difficulty breathing to assess whether the respiratory rate (RR) is high enough for a particular age to prescribe antibiotics and treat suspected pneumonia. This is defined by the World Health Organization (WHO) Integrated Management of Childhood Illness (IMCI) guidelines [3] for FLHFWs and the Integrated Community Case Management (iCCM) guidelines for CHWs [4]. Current standard practice for frontline health workers is to count RR manually by observing chest movements for 60 seconds. In practice defining a breath and counting RR can be difficult, as children breathe irregularly and faster than adults and the child may not be calm and still for a full minute. Misclassification of the observed rate remains high [5,6], often leading to inappropriate treatment [7].

The Acute Respiratory Infection Diagnostic Aids (ARIDA) project [8] was initiated as a response to the call for better pneumonia diagnostic aids [9,10]. A target product profile (TPP) was shared with industry, academia, and partners to encourage and guide development of new automated RR counting devices [11]. The ARIDA technical specification listed in the United Nations Children’s Fund (UNICEF) request for proposals outlines that any ARIDA must automatically detect and display the RR to aid in the classification of suspected pneumonia in children from the age of 0 to 59 months and include a visual indicator for notification of above or below the age-specific fast breathing thresholds as defined by the WHO IMCI guidelines [3].

**Figure 1:**
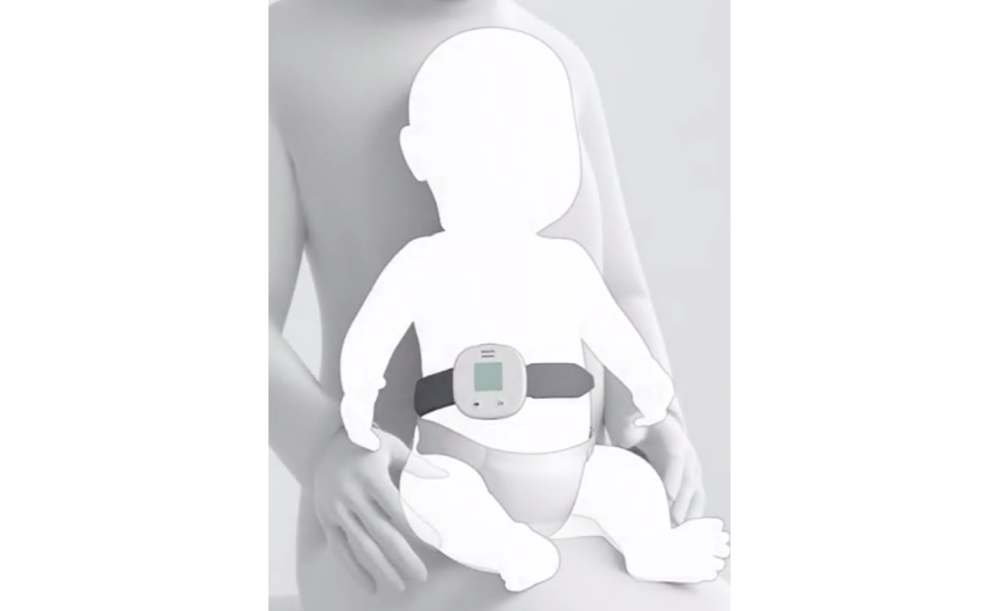
Illustration of the Philips ChARM device positioned on a child.

In response to the TPP, Philips developed the Children’s Respiration Monitor (ChARM) device, which uses an accelerometer-based system to measure the RR in children 0 to 59 months and automatically classifies the breathing rate as fast or normal, based on the age of the child. ChARM is intended to be used by CHWs in low-resource settings. It is strapped around the belly of the child using an elastic belt (Figure 1). ChARM is the first product to be tested as part of the ARIDA field trials, implemented by the Malaria Consortium in Ethiopia and Nepal and sponsored by UNICEF in partnership and with funding from “la Caixa” Banking Foundation.

In Ethiopia, pneumonia is the number one cause of under-5 mortality, responsible for 16% deaths in children aged younger than 5 years in 2016 [12]. Ethiopia has scaled up iCCM of childhood illness in all regions following a national policy change supporting community-based treatment of childhood pneumonia by CHWs (locally known as health extension workers [HEWs]) in early 2010 [13]. As part of Ethiopia’s HEW program, over 42,000 HEWs have been trained for 1 year in iCCM and equipped to assess, classify, and manage pneumonia, malaria, diarrhea, and severe acute malnutrition and provide preventive and curative health services [13]. This paper presents the study design for the evaluation of agreement between an ARIDA and a reference standard RR count for children in Ethiopia.

## Methods

### Study Aims and Objectives

This study aims to understand whether an ARIDA RR count agrees with an expert panel of pediatricians counting RR by reviewing a video of the child’s chest for 60 seconds (reference standard) for children aged younger than 5 years with cough and/or difficult breathing.

The primary objective of this study is to determine the performance of an ARIDA, as defined by agreement and consistency, in children aged younger than 5 years with cough and/or difficulty breathing. The secondary objective is to determine the performance of expert clinicians (ECs) counting RR, as defined by agreement and consistency, in children aged younger than 5 years with cough and/or difficulty breathing. The third objective is to measure RR fluctuation over time after ARIDA device attachment in normal breathing children aged 2 to 59 months.

### Study Design

The study is a cross-sectional study comprising three types of RR evaluations: agreement, consistency, and RR fluctuation over time.

### Study Site

The study was conducted in the pediatric in- and outpatient departments at Saint Paul’s Hospital and Millennium Medical College in Addis Ababa, Ethiopia. This hospital was selected based on the high incidence of pneumonia in outpatient and inpatient departments, interest and willingness of hospital managers to host the study, availability of Integrated Management of Neonatal and Child Illness (IMNCI)-trained [14] expert clinicians (ECs), and availability of a suitable study room, reliable electricity supply, and access to treatment including amoxicillin and oxygen.

### Ethics Approval and Consent to Participate

Ethical approval was obtained from the Armauer Hansen Research Institute/ALERT Ethics Review Committee (a biomedical research institute in Ethiopia) on March 7, 2017 (ref. PO02/17) and favorable ethical opinion received by the Liverpool School of Tropical Medicine Ethics Committee. All participants consented to the study by reading and signing the information and consent form.

### Study Participants

All children attending in- and outpatient departments at Saint Paul’s Hospital and Millennium Medical College in Addis Ababa between April 5 and May 22, 2017, were potential participants in the study and were systematically screened for eligibility. Children aged 0 to <2 months were excluded from the consistency evaluation due to the anticipated difficulty in attaching two devices at once to a small child. Children aged 0 to <2 months and those with fast breathing were excluded from the fluctuation evaluation due to anticipated difficulty in measuring RR in this group for an extended period of time and also to isolate the effect of the ChARM attachment on RR from other causes of RR fluctuation. All other children aged younger than 5 years who were accompanied by a caregiver aged 18 years and older, not too agitated to be assessed by a research nurse, who did not present with general danger signs or IMNCI referral signs or device manufacturer safety exclusion criteria (wearing supportive device at area of chest/belly, skin not intact in chest/belly, born before 37 weeks of gestation [<2 months only]), were not an inpatient being managed by barrier nursing (such as severe burns, child with neutropenia, severe infectious diseases), and were not advised against research procedures by the supervising clinician were eligible to participate in the study.

General danger signs for newborns (<28 days) include active convulsions or fits, not feeding well, fever (37.5℃ [99.5℉] or above), low body temperature (35.5℃ [95.9℉] or below), movement only when stimulated, or no movement even when stimulated and for all other age groups include lethargy or unconsciousness, not able to drink/breastfeed, vomiting everything, and active convulsions or fits. IMNCI referral signs for all children include stridor in a calm child, chest indrawing, severe dehydration, severe persistent diarrhea, very severe febrile disease, severe complicated measles, mastoiditis, complicated severe malnutrition, and severe anemia. Written informed consent was obtained from the caregiver before enrollment. Two ECs with extensive experience in assessing and treating children with suspected pneumonia using IMNCI guidelines were selected. They were required to have BSc nursing qualification and an IMNCI certificate.

### Outcome Measures

The primary outcome for the agreement evaluation was the mean difference in RR between ARIDA and the reference standard, which summarized the lack of agreement by calculating the average deviation between measures. Similarly for the consistency evaluation, the mean difference in RR between two ARIDA devices was calculate. Table 1 shows all the outcome measures for the study by objective.

- Primary objective: determine the performance of the ARIDA device as defined by agreement and consistency in children aged younger than 5 years with cough or difficulty breathing in a controlled setting
- Secondary objective: determine the performance of ECs counting RR as defined by agreement and consistency in children aged younger than 5 years with cough or difficulty breathing in a controlled setting
- Third objective: measure RR fluctuation over time after ARIDA device attachment in normal breathing children aged 2 to 59 months in a controlled setting

**Table 1:** Primary objective and outcome measures.

Outcomes		Objective		Evaluation	
**Primary**					
	Mean difference between ARIDA^a^ and VEP^b^ RR^c^ count		1		ARIDA agreement
	Mean difference between RR counts from two ARIDA devices started simultaneously		1		ARIDA consistency
**Secondary**					
	RMSD^d^ between ARIDA and VEP RR counts		1		ARIDA agreement
	Percentage ARIDA RR counts ±2 breaths from VEP RR count		1		ARIDA agreement
	Absolute mean difference between ARIDA and VEP RR count		1		ARIDA agreement
	Positive and negative percentage agreement between ARIDA and VEP RR classification		1		ARIDA agreement
	Percentage unsuccessful attempts with ARIDA		1		ARIDA agreement
	Percentage failures (3 unsuccessful attempts) with ARIDA		1		ARIDA agreement
	Mean time taken to get an ARIDA RR count		1		ARIDA agreement
	RMSD between RR counts from two ARIDA devices started simultaneously		1		ARIDA consistency
	RMSD of time taken to get ARIDA RR count for two ARIDA devices started simultaneously		1		ARIDA consistency
	Mean difference between EC^e^ and VEP RR count		2		EC agreement
	Mean difference between RR counts from two simultaneous ECs		2		EC consistency
	RMSD between RR counts from two simultaneous ECs		2		EC agreement
	Percentage EC counts ±2 breaths from VEP RR count		2		EC agreement
	Absolute mean difference between EC and VEP RR count		2		EC agreement
	Positive and negative percentage agreement between EC and VEP RR classification		2		EC agreement
	Percentage unsuccessful attempts for EC RR count		2		EC agreement
	Percentage failures (3 unsuccessful attempts) for EC RR count		2		EC agreement
	RMSD between RR counts for two simultaneous ECs		2		EC consistency
	Difference between RR at baseline and after 1, 3, and 5 minutes after ARIDA attachment		3		Fluctuation
	RR trend plotted on a line graph before (baseline) and 1, 3, and 5 minutes after ARIDA attachment		3		Fluctuation

^a^ARIDA: Acute Respiratory Infection Diagnostic Aid (Philips Children’s Respiration Monitor device).

^b^VEP: video expert panel (reference standard).

^c^RR: respiratory rate.

^d^RMSD: root mean squared difference.

^e^EC: expert clinician.

### Data Collection and Management

Data were collected using an electronic data collection platform (CommCare, Dimagi) installed onto password-protected 7C Pro tablets (Tecno Mobile) and backed up to a protected cloud server. Four-digit unique identification codes were used to anonymize patient data. All videos were transferred using password-protected external hard drives, and paper consent forms were stored in opaque carriers in locked cabinets. All RR evaluation data were entered by two independent research assistants. The data manager downloaded data daily and entered it into a data checker with in-built validation checks. Source videos showing the ARIDA device with the RR count displayed were used to verify ARIDA counts should the two research assistants disagree. Other inconsistencies were rectified by tracing back to paper data entry forms or querying the counts directly with the research team.

### Training and Pretest

The video expert panel (VEP) members and ECs were trained for two days on the WHO IMCI method to count RR including practice for half a day using videos of known RR counts [3]. They were individually evaluated using different RR videos to ensure that they were able to count RR ±2 breaths per minute (bpm) from the known RR in 4 out of 5 training videos [15]. All VEP members and ECs passed the competency assessment before starting video review.

Following training, an 8-day pretesting of procedural activities including patient screening, patient flow, and data collection was conducted to ensure the research team was conversant with the data collection procedures, devices, and videography equipment to be used in the study. There was also a pretest of the video panel reference standard evaluations and refresher training on general danger signs, breath-counting, signs of stridor, and chest indrawing with the IMNCI training video.

### Evaluations

Patients were screened by a research nurse in the in- and outpatient departments of the hospital using a screening checklist to ascertain the child’s eligibility. An RR classification was made during prescreening by the research nurse using the Mark 2 Acute Respiratory Infection (MK2 ARI) timer to determine whether the child had fast or normal breathing. The prescreening assessment was conducted in a separate part of the hospital and not communicated to the ECs to blind them to the RR classification. Children were enrolled prospectively based on eligibility determined by the screening procedure. The research team then decided, based on the state of the child, age, and breathing status, how many elements of the study to conduct on each child—agreement, consistency, and/or RR fluctuation. Table 2 shows the number of participants aimed to be enrolled to each element of the study by age group and breathing status.

**Table 2:** Enrollment targets for each type of evaluation by age group and breathing status.

Age group and breathing status (based on screening)			Enrollment targets for three evaluations				
			Agreement		Consistency		Fluctuation
**0 to <2 months**							
	Normal	13		—		—	
	Fast	39		—		—	
**2 to <12 months**							
	Normal	13		13		15	
	Fast	39		39		—	
**12 to 59 months**							
	Normal	13		13		15	
	Fast	39		39		—	

For the agreement evaluation, the research assistant attached an ARIDA to the child and ensured the child was positioned correctly according to device instructions: with his/her back fully supported, either on the lap of the caregiver or lying down on a bed, and the device in line with the child’s belly button and one of the nipples [16]. Once the child was calmed, usually by the research assistant clicking their fingers, the videographer started recording and the ARIDA and EC RR count started simultaneously. The EC was blinded to the ARIDA RR count by placing ARIDA on the far side of the child’s belly and using a paper cover to shield the screen. The time taken to get an ARIDA count (from when the OK button was pressed to when the device beeped to signal completion of the RR count) was also obtained by a research assistant using a stopwatch. After 60 seconds, if the EC had not obtained an RR count, the EC attempt was recorded as unsuccessful and repeated for both the EC and ARIDA. After 5 minutes or if the ARIDA displayed an error message, the ARIDA attempt was recorded as unsuccessful (with a reason) and repeated for both EC and ARIDA. If the third attempt was still unsuccessful for either device or EC, the evaluation was recorded as a failure. Fifteen different ARIDA devices were used and rotated systematically for all evaluations.

The consistency evaluation followed the same procedure as the agreement evaluation with two ARIDA devices attached to a child using a single belt, positioned in line with each nipple and measured RR from the same starting point. Time taken to obtain each ARIDA RR count was recorded by two research assistants using stopwatches. To measure the consistency between ECs, two ECs conducted separate manual RR counts with MK2 ARI timers over an identical 60-second period. For an EC or ARIDA attempt to be successful, both ECs or both ARIDA devices had to get an RR count. For the RR fluctuation evaluation, an EC counted RR with the MK2 ARI timer for 60 seconds. Following this, the ARIDA was attached to the child and the EC did three more RR counts for 60 seconds in the following time periods: 0 to 1 minute, 2 to 3 minutes, 4 to 5 minutes. On completion of the evaluation, the research team debriefed the caregiver and ensured medical management for the child was completed by the relevant hospital staff.

### Reference Standard

The reference standard for the agreement evaluation was a video review by two to four independent VEP members. They were all practicing pediatricians with over 5 years’ experience managing pneumonia in children aged younger than 5 years and who had received refresher training in counting RR as per WHO IMCI guidelines (3).

First, two VEP members independently watched a video of the child’s chest movements, edited with the layover of the ChARM start and stop sound and a 60 second timer, and counted the number of breaths observed in a full minute. Beep sounds were added by the videographer in sync with the original sounds made by the ChARM device at the start (when the start button on ChARM is pushed) and at the end (when the ChARM displays the result). The sound recorded at the time of recording was also muted to allow the VEP to focus on the sound of the start and stop beeps.

If the first two VEP members agreed (≤±2 bpm), a mean RR count was used as the reference standard. If they disagreed (>±2 bpm), a third VEP member reviewed the video and if two out of three counts agreed (≤±2 bpm), the mean of the two closest RR counts was used. If all three VEP members disagreed (>±2 bpm), the video was sent for review to a fourth VEP member. If the fourth VEP member’s count agreed (≤±2 bpm) with any of the first three VEP members’ counts, the mean of the two closest counts was used. If all four panel members disagreed (>±2 bpm), the data from this evaluation were excluded from the agreement analysis.

### Sample Size

The primary outcome on which sample size was based was the agreement between the ARIDA and VEP RR counts. As per Bland-Altman [17], we conducted a precision-based sample size calculation based on the confidence interval for the 95% limits of agreement. The formula estimates the required number of children per age group (n) based on the desired width of the confidence interval. Using normal approximation and allowing a confidence interval of ±0.5 standard deviations of the difference between the two devices, a sample size of 46 children per age group was required for the agreement and consistency evaluations, adjusted to 52 per group for failure to get a reference standard count. For the RR fluctuation evaluation, a sample size of 30 children was used.

### Data Analysis

Data analysis for all three RR evaluations was conducted in Stata 13 (StataCorp LLC) and Excel (Microsoft Corp). First, the number of children screened, eligible, consented, and enrolled in each type of evaluation was described. Baseline characteristics (age and sex) by screening breathing status (normal/fast) for those enrolled were described. All full-length source videos were reviewed for quality assurance purposes, and descriptive information on the video quality was recorded, including those where all four VEP members disagreed on the RR count. For the ARIDA and EC agreement and consistency evaluations, mean difference, root mean square difference, absolute mean difference, proportion of RR counts ±2 bpm from the reference standard, and positive and negative percentage agreement with 95% confidence intervals were calculated in Stata 13 by age group, and Bland-Altman plots with limits of agreement and 95% confidence intervals by age group and breathing status were created. Percentage of unsuccessful attempts and failures (defined by three unsuccessful attempts) and mean time to get an ARIDA RR count were calculated. A per-protocol analysis was used whereby children were excluded from the analysis if an RR could not be obtained simultaneously by the ARIDA and by the EC, with a VEP RR reading where at least two of the panel members were within ±2 bpm of each other. For the RR fluctuation evaluation, mean difference in the RR count between baseline and 1 minute, 1 and 3 minutes, and 3 and 5 minutes were calculated. The proportion of children with fast or normal RR classification at baseline and the change between RR classifications over time were analyzed.

### Quality Assurance, Supervision, and Monitoring

Malaria Consortium and UNICEF (Supply Division and Ethiopia Country Office) conducted quality assurance visits every 2 weeks to the research site during data collection. All data collected from the screening and RR evaluations were checked and verified by the data manager daily. A sample of three videos was sent weekly to an independent study advisor for RR evaluation using WHO IMCI guidelines [3] and to Malaria Consortium HQ for quality assurance. The project had an 11-person Advisory Committee made up of experts on maternal and child health who provided technical oversight and reviewed the study protocol.

## Results

The project was funded in 2016. Data were collected between 5 April until 22 May 2017. Authors are drafting the results for publication.

## Discussion

Accurately diagnosing pneumonia in children aged younger than 5 years remains a significant problem in resource-poor settings. Manually counting RR is inherently challenging for CHWs, resulting in both over and under diagnosis and treatment. This diagnostic performance study in Ethiopia aims to provide evidence for the performance of an ARIDA device when used in a controlled setting.

Evaluating performance of new RR counters is difficult due to the absence of an appropriate gold standard. Selecting a robust reference standard when designing this study was a challenge. The aim was to have one reference standard for evaluating any new ARIDA regardless of the technology for calculating RR. Retrospective review of video recordings by a panel of experts has been used as a reference standard for a number of pneumonia studies [18-20]. It allows many experts to assess the same patient, thus limiting bias that could arise from having one expert per child and reducing the number of experts present in the room, whose presence could agitate the child and affect their RR. It also allows the expert to review the evaluation numerous times and adjust the speed and zoom of the video to aid the counter. An interrater agreement study in northeast Tanzania measured the agreement between two pediatricians reviewing RR videos of children aged 2 to 59 months. They found that in two-thirds of cases, both pediatricians agreed on the RR within ±2bpm, which represents fair agreement (kappa=.34) and in ninety-six percent of cases, both pediatricians agreed on RR classification, representing perfect agreement (kappa=.85) [21]. Recognizing the limitations of humans counting RR using a video, in the absence of a gold standard and with recommendations from the Advisory Committee, the video reference standard was selected.

A strength of this study is that the design allows for contemporaneous comparison between the RR count from the ARIDA, EC, and video reference standard. While the comparison is imperfect, as the ARIDA can take longer than 60 seconds to obtain a count compared with the VEPs and ECs who assessed RR over 60 seconds, it remains useful for identifying increased RR and therefore whether the RR classification of breathing status is comparable.

To minimize RR counting errors, this study was implemented with two days of training and an assessment for the VEP members and ECs to ensure a consistent methodology for RR counting. Interrater agreement between humans could be improved with guidance about how to define a breath versus a movement and additional standardization between humans through training and practice using this guidance to count RR for a selection of videos. Furthermore, a video annotation aid that allows the panel member to mark breaths and non–breath movements directly on the video could reduce human RR counting inconsistencies and allow for discussion and consensus building between panel members about the RR of videos.

Mean difference with 95% confidence intervals was selected as the agreement measure for the primary outcome. A disadvantage of this measure is that positive and negative bias cancel out to give a lower mean difference. An alternative is to use the limits of agreement with 95% confidence intervals on the Bland-Altman plot to visually show the agreement between the two measures and estimate the precision of the estimates. We recommend that global consensus and guidance on an acceptable level of agreement between a new automated RR counter and a reference standard as measured by the range of the limits of agreement on a Bland-Altman plot is sought in addition to global consensus on the reference standard methodology. This will provide ministries of health with evidence to make an informed decision on the performance of new RR devices to inform introduction and scale up of these devices.

## Abbreviations

ARI: acute respiratory infection
ARIDA: Acute Respiratory Infection Diagnostic Aid
bpm: breaths per minute
ChARM: Children’s Respiration Monitor
CHW: community health worker
EC: expert clinician
FLHFW: first-level health facility worker
HEW: health extension worker
iCCM: Integrated Community Case Management
IMCI: Integrated Management of Childhood Illness
IMNCI: Integrated Management of Neonatal and Child Illness
RR: respiratory rate
TPP: target product profile
UNICEF: United Nation Children’s Fund
VEP: video expert panel
WHO: World Health Organization
